# Thermodynamic studies of solute–solute and solute–solvent interactions in ternary aqueous systems containing {betaine + PEGDME_250_} and {betaine + K_3_PO_4_ or K_2_HPO_4_} at 298.15 K

**DOI:** 10.1038/s41598-023-43906-0

**Published:** 2023-10-18

**Authors:** Mohammed Taghi Zafarani-Moattar, Hemayat Shekaari, Soheila Asadollahi

**Affiliations:** Department of Physical Chemistry, University of Tabriz, Tabriz, Iran

**Keywords:** Chemistry, Physical chemistry, Pharmaceutics

## Abstract

In this work, to evaluate solute–solute, solute–solvent and phase separation in aqueous systems containing {betaine + poly ethylene glycol dimethyl ether with molar mass 250 g mol^−1^ (PEGDME_250_)}, {betaine + K_3_PO_4_} and {betaine + K_2_HPO_4_}, first water activity measurements were made at 298.15 K and atmospheric pressure using the isopiestic technique. The water iso-activity lines of these three systems were obtained which have positive deviations from the semi-ideal solutions. This suggests that betaine-polymer and betaine-K_3_PO_4_ or betaine-K_2_HPO_4_ interactions are unfavorable; and these mixtures may form aqueous two-phase systems (ATPSs) at certain concentrations. Indeed the formation of ATPSs was observed experimentally. Then, osmotic coefficient values were calculated using the obtained water activity data; and, using the polynomial method the solute activity coefficients were determined. Using these activity coefficients, the transfer Gibbs energy ($\Delta {G}_{tr}^{i}$) values were calculated for the transfer of betaine from aqueous binary to ternary systems consisting polymer (PEGDME250) or salts (K_3_PO_4_ and K_2_HPO_4_). The obtained positive $\Delta {G}_{tr}^{i}$ values again indicated that there is unfavorable interaction between betaine and these solutes. Finally, the volumetric and ultrasonic studies were made on these systems to examine the evidence for the nature of interactions between betaine and the studied salts or polymer.

## Introduction

From environmental view point use of nontoxic and renewable compounds is an important issue that has been attempted recently by many research workers on green chemistry. To replace or at least reduce the use of hazardous materials with benign ones in production or extraction processes is considered to be a vital goal to sustainable development^1^.

Recently, betaine as an environmentally safe material has received more attention by some research workers^2,3^. Betaine is known as a non-toxic, biodegradable natural material with the obvious name for 2-(trimethylazaniumyl) acetate which is the derivative of the amino acid glycine. It is extracted as a by-product from sugar beet; and widely used as a feed additive and as an osmolyte^4^. The osmolyte property of betaine is related to its dipolar zwitterions structure which dissolves easily in water. In addition to the mentioned properties, betaine is a methyl group donor and can be taken part in transemethylation reactions and it acts as an organic osmolyte in protecting cells under stress. Finally, due to the methylating and amino acid properties it participates in energy and protein metabolism^5^.

Design and development of new green processes requires the study of thermodynamic properties of the system. To contribute to a better knowledge of thermodynamic behavior of the system under investigation experimental phase equilibrium data is needed. These data enable us to calculate osmotic and activity coefficients in mixtures which can be used for obtaining some information regarding the solute–solvent interactions and development of reliable thermodynamic models. The obtained results are important in regard with possible two-phase formation of these kinds of ternary systems which may have applications in partitioning and extraction of drugs or proteins^6^. Recently, using these kinds of ternary systems partitioning studies has been made on some proteins^6^. However, further studies regarding the solute–solvent interactions, two-phase formation behavior and partitioning of biomaterials is needed.

Vapor pressure data for betaine systems are extremely scarce and many studies are needed. Therefore, in this work, first, water activity for ternary {betaine + poly ethylene glycol dimethyl ether with molar mass 250 g mol^−1^ (PEGDME_250_) + water}, {betaine + K_3_PO_4_ + water} and {betaine + K_2_HPO_4_ + water} systems were measured at 298.15 K to evaluate the possible interactions between these components. There is no report on the water activity or vapor pressure for these systems; and only binodal curves for the two latter systems containing potassium salts and extraction of proteins in the corresponding aqueous two-phase systems have been reported^6^. In this work, similar vapor–liquid measurements were also carried out on the aqueous binary betaine solutions for which no data is available in open literature. There are several methods for obtaining osmotic coefficient or activity such as electromotive force method, headspace chromatography and vapor pressure osmometry^7^. In the present study, we have used the isopiestic technique which is an accurate method to determine solvent activity of solutions^8,9^. Since, quality of isopiestic measurements depends mainly on the temperature equality between the solutions, keeping the whole system at a constant temperature is required. In addition to reporting some thermodynamic properties of the mentioned mixtures, we attempted to provide solute–solute and solute–solvent interactions by calculating the solute activity coefficients and the transfer Gibbs energy ($\Delta {G}_{tr}^{i}$) values for the transfer of PEGDME_250_, K_3_PO_4_ and K_2_HPO_4_ from binary to ternary aqueous solutions containing betaine using the measured water activity data and polynomial method^10,11^. Furthermore, to confirm the solute–solute interactions results deduced from water activity treatment, volumetric and ultrasonic properties such as the limiting apparent molar volume (${V}_{\varphi }^{0}$), transfer apparent molar volume (${\Delta }_{tr}{V}_{\varphi }^{0}$), and also transfer apparent isentropic compressibility (${\Delta }_{tr}{k}_{\varphi }^{0}$) of polymer and salts from binary to ternary aqueous solutions containing betaine were determined from the corresponding measured density and speed of sound data.

This work presents the thermodynamic study of the material betaine which is in popular demand in scientific research from environmental point of view. The obtained thermodynamic data with experimental verification will stands as a reference in helping the designing and developments in green chemistry approaches and in the development of thermodynamic systems utilizing betaine. In particular, the use of betaine as a component of two-phase system is very important in extraction of biomaterials. Also, since there is no information regarding the water activity and vapor pressure of betaine in the literature, these data stands as a reference for future studies.

## Experimental

### Materials

The betaine was obtained from Jinan Grace Industry (> 98%w/w).The salts K_3_PO_4_ (> 99.5%w/w), K_2_HPO_4_ (> 99.5%w/w) and NaCl (> 99.5%w/w) were supplied by Merck. All materials were dried in vacuum and kept over P_2_O_5_ before used. The water contents of materials were measured by a Karl-Fischer titrator (751 GPD Titrino-Metrohm, Herisau, Switzerland). In preparing the sample solutions, the measured water contents was accounted and double distilled water was applied. The specifications for the used chemicals are listed in Table 1.

**Table 1:** Descriptions of the used chemicals.

Material	Provenance	CAS number	Purity (mass fraction)^*a*^	Molecular formula	Water content (mass fraction)^b^
Betaine	Jinan grace industry	107-43-7	> 0.98	C_5_H_11_NO_2_	0.0008
Sodium chloride	Merck	57-48-7	> 0.995	NaCl	0.0007
Tri-potassium phosphate	Merck	57-50-1	> 0.995	K_3_PO_4_	0.0009
Dipotassium hydrogen phosphate	Merck	7647-14-5	> 0.995	K_2_HPO_4_	0.0003
Poly ethylene glycol di-methyl ether 250	Merck	24,991-55-7	> 0.995	CH_3_O(CH_2_CH_2_O)_n_CH_3_	0.0003

^a^As stated by the supplier.

^b^Water contents were determined by Karl-Fischer method.

### Apparatus and procedure

#### Vapor – liquid equilibrium measurements

In this study, for obtaining water activity and vapor pressure at 298.15 K the improved isopiestic apparatus was applied^8,9^. In measuring the water activity for binary aqueous betaine solutions, five leg manifold was used in which for each of pure betaine and standard pure NaCl, two flasks are employed and the central flask was considered for water supply. Also, this procedure with a seven leg manifold was employed in the case of water activity measurements for (betaine + PEGDME_250_ + water), (betaine + K_3_PO_4_ + water) and (betaine + K_2_HPO_4_ + water). In this case, for each of pure betaine, pure salt (K_3_PO_4_/K_2_HPO_4_) or polymer one flask was used. Two flasks were poured with standard sodium chloride. The remaining two flasks of the manifold are considered for {betaine + K_3_PO_4_/ K_2_HPO_4_} or {betaine + PEGDME_250_}. Before doing anything, it was necessary to degas and remove the air of the solutions which was made by evacuating the apparatus slowly and frequently. To reach the equilibrium between solutions, the apparatus was slowly immersed in a bath for about 120 h. The temperature controller with uncertainty of 0.01K was used. Then, the apparatus was removed from the bath and the mass of each flask and therefore solute mass fractions were determined with an analytical balance with a precision of ± 1 × 10^–7^ kg. We used the differences between mass fractions of two NaCl solutions as an equilibrium criteria; so that the equilibrium is assumed when this difference is less than 0.1%. In isopiestic equilibrium vapor pressure and water activity in the sample solutions and reference are the same. This enables us to calculate the osmotic coefficient for the reference solution, from which the osmotic coefficient of the each sample in isopiestic equilibrium with the standard NaCl is readily determined. In this work, for the required osmotic coefficient values of the reference solutions the correlation relation suggested by Colin et al.^12^ was considered.

#### Density and speed of sound measurement

For volumetric and ultrasonic studies, {betaine + PEGDME_250_ + water}, {betaine + K_3_PO_4_ + water} and {betaine + K_2_HPO_4_ + water} solutions at different molalities of betaine as a solute and different molality of {polymer or salt + water} as a solvent (i.e. 0.1, 0.2, 0.3) mol kg^−1^ were prepared in glass vessels and the corresponding density $(\rho )$ and speed of sound $(u)$ values were measured with (Anton Paar DSA 5000 densitometer and speed of sound analyzer) at *T* = 298.15 K and atmospheric pressure $( \approx 85KPa)$.

The uncertainty values of 0.15 kg m^−3^ and 0.5 m s^−1^ were estimated for density and speed of sound, respectively. Before doing anything, it was necessary to calibrate densitometer device; for this purpose dried air and double distilled water were used. The accurate temperature controller built in the apparatus enable us to carry out these measurements within ± 0.01 K which is a required condition for such measurements^13^.

## Result and discussion

### Experimental results

#### Water activity results

To study the vapor–liquid equilibrium behavior of binary aqueous solutions of betaine and ternary aqueous betaine solutions containing the salts (K_3_PO_4_/K_2_HPO_4_) or PEGDME_250_, water activity measurements were carried out at 298.15 K using isopiestic method^8,9^. Considering equal water activity of standard and sample solutions at isopiestic equilibrium, and relation 1, the osmotic coefficient, $\phi$ of the solution can be calculated^14^:

1 $$\phi = \frac{{v_{R} \phi_{R} m_{R} }}{vm}$$

In Eq. (1), *m* and $m_{R}$ stand for the isopiestic equilibrium molalities (mol kg^−1^) of the sample and reference (NaCl) solutions, respectively;$\phi_{R}$ is the osmotic coefficient of the reference solution, $\nu_{R}$ and $\nu$ denote respectively the sum of stoichiometric numbers of the anion and cation in the reference and the salts (K_3_PO_4_/K_2_HPO_4_) or PEGDME_250_, solutions. The osmotic coefficient values calculated from Eq. (1) can be used along with Eqs. (2) and (3) to obtain the activity of water and the vapor pressure *P* in polymer and salt solutions^14^.

2 $$\ln a_{W} = - \phi \nu mM_{W}$$

3 $$Lna_{W} = \ln \left( {\frac{P}{{P_{W}^{0} }}} \right) + \frac{{(B - V_{W}^{0} )(P - P_{W}^{0} )}}{RT}$$

Here, *M*_W,_ $V_{W}^{0}$ and $P_{W}^{0}$ are used respectively for the molar mass, molar volume and vapor pressure of pure water. *B* stand for second virial coefficient of water vapor and its value at *T* = 298.15K was obtained from the Rard and Platford equation^15^. *R* is the gas constant and *T* is the absolute temperature. The Kell equation^16^ and the equation of state proposed by Saul and Wagner^17^ were used respectively to determine $V_{W}^{0}$ and $P_{W}^{0}$. After the system reaches isopiestic equilibrium, *a*_w_ and *P* values were calculated using Eqs. (2) and (3), respectively.

**Figure 1:**
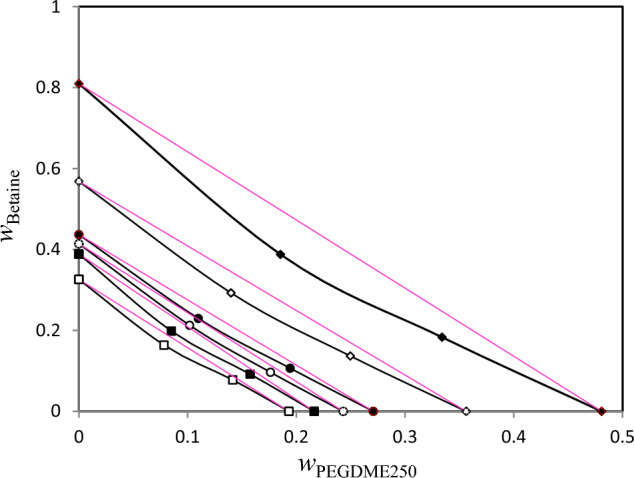
Isoactivity line (*a*_w_) for ternary solution{Betaine + PEGDME_250_ + H_2_O} with respect semi ideal lines (red lines) at *T* = 298.15 K. Mass fraction of betaine (*w*_Betaine_) plotted against mass fraction of PEGDME_250_ (*w*_PEGDME250_): “filled diamond”, 0.9623; “diamond”, 0.9390; “filled circle”, 0.9343; “circle”, 0.9199; “filled square”, 0.8692; “square”, 0.7207.

**Figure 2:**
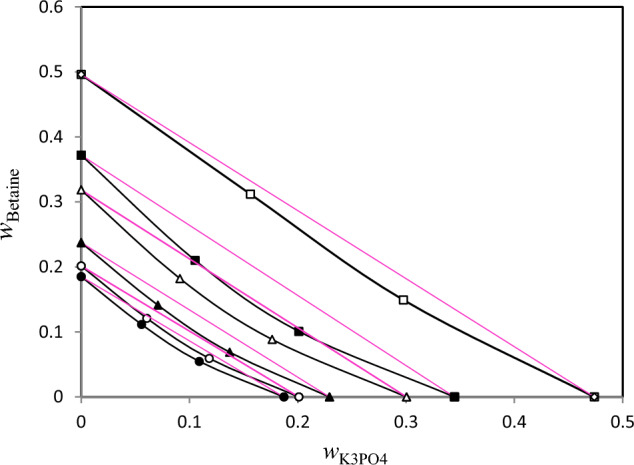
Isoactivity line (*a*_w_) for ternary solution{Betaine + K_3_PO_4_ + H_2_O} with respect semi ideal lines (red lines) at *T* = 298.15 K. Mass fraction of betaine (*w*_Betaine_) plotted against mass fraction of K_3_PO_4_ (*w*_K3PO4_): “square”, 0.9545; “filled square”, 0.9491; “triangle”, 0.9374; “filled triangle”, 0.9035; “circle”, 0.8791; “filled circle”, 0.6942. The red dotted lines are semi ideal lines.

**Figure 3:**
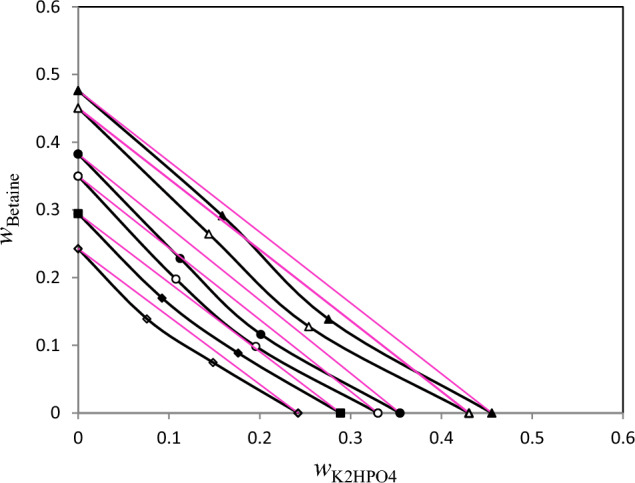
Isoactivity line (*a*_w_) for ternary solution {Betaine + K_2_HPO_4_ + H_2_O} with respect semi ideal lines (red lines) at *T* = 298.15 K. Mass fraction of betaine (*w*_Betaine_) plotted against mass fraction of K_2_HPO_4_ (*w*_K2HPO4_): “filled triangle”, 0.9304; “triangle”, 0.9279, “filled circle”, 0.8997; “circle”, 0.7931; “filled diamond”, 0.7063; “diamond”, 0.5433. The red dotted lines are semi ideal lines.

The for binary (betaine + water) solutions, the values of water activity, osmotic coefficient and vapor pressure are collected in Table 2. In Tables 3, 4, 5, water activity values are given for (betaine + PEGDME_250_ + water), (betaine + K_3_PO_4_ + water) and (betaine + K_2_HPO_4_ + water), and the corresponding iso-activity lines are shown in Figs. 1, 2, 3, respectively. In each of presented lines in these Figures, the four points have the same water activity and therefore the same chemical potential.

**Table 2:** Mass fractions (*w*) in isopiestic equilibrium, water activity (*a*_W_), osmotic coefficient (${\phi }_{R}$) and vapor pressure (*p*) for the {betaine (1) + H_2_O (2)} system at *T* = 298.15 K and *P* ≈ 85 kPa^a^.

^*b*^*w*_1_	^*c*^*m*_1_(mol∙kg^−1^)	*w*_NaCl_	*m*_NaCl_(mol∙kg^−1^)	*ϕ*_R_	*a*_w_	*p *(kPa)
0.4145	6.0598	0.1879	3.9342	1.453	0.8535	2.708
0.4091	5.9131	0.1840	3.8641	1.455	0.8565	2.717
0.3740	5.2110	0.1650	3.2450	1.334	0.8824	2.800
0.3434	4.4646	0.1408	2.7801	1.295	0.9012	2.860
0.3038	3.7251	0.1170	2.2670	1.227	0.9211	2.924
0.2817	3.3494	0.1044	1.9943	1.181	0.9313	2.956
0.2366	2.6476	0.0891	1.6739	1.231	0.9433	2.994
0.2301	2.5514	0.0856	1.5820	1.201	0.9463	3.003
0.1956	2.1031	0.0824	1.3461	1.225	0.9557	3.032
0.1371	1.6558	0.0771	1.1090	1.267	0.9633	3.057
0.1011	1.4590	0.0621	1.0541	1.363	0.9658	3.064

^a^Standard uncertainties *u* for temperature, pressure, mass fraction, and molality are *u(T)* = 0.1 K, *u*(*P*) = 0.01 kPa, *u*(*w*) = 0.002, and *u(m)* = 0.003 mol kg^−1^, respectively with confidence level of 0.95. The combined standard uncertainty of vapor pressure, water activities, and the osmotic coefficient are *u* (*p*) = 0.02 kPa, *u* (*a*_w_) = 0.002, and *u* (*ϕ*_R_) = 0.02, respectively with confidence level of 0.68.

^b^Mass fraction of betaine.

^c^Molality of betaine.

**Table 3:** Mass fractions (*w*) in isopiestic equilibrium, water activity (*a*_W_), osmotic coefficient (${\phi }_{R}$) and vapor pressure (*p*) for the {betaine (1) + PEGDME_250_ (2) + H_2_O (3)} system at *T* = 298.15 K and *P* ≈ 85 kPa^a^.

^*b*^*w*_1_	$\frac{{m}_{1}}{{\mathrm{mol}\cdot \mathrm{kg}}^{-1}}$	^*c*^*w*_2_	${m}_{2}/{\mathrm{mol}\cdot \mathrm{kg}}^{-1}$	${w}_{NaCl}$	${m}_{NaCl}/{\mathrm{mol}\cdot \mathrm{kg}}^{-1}$	${a}_{w}$	${\phi }_{R}$	$p/\mathrm{kPa}$
0	0	0.326	1.9344	0.0617	1.1258	0.9623	0.946	3.055
0.0783	0.882	0.1638	0.8646					
0.1416	1.5479	0.0776	0.3978					
0.1932	2.0435	0	0					
0	0	0.3887	2.5435	0.0944	1.7835	0.939	0.98	2.981
0.095	1.147	0.1985	1.1241					
0.1576	1.9313	0.0919	0.4963					
0.2164	2.6429	0	0					
0	0	0.4141	2.8265	0.1005	1.9122	0.9343	0.987	2.966
0.1018	1.267	0.2128	1.2417					
0.1762	2.0688	0.0966	0.5316					
0.2431	2.7413	0	0					
0	0	0.4367	3.1007	0.1183	2.296	0.9199	1.01	2.92
0.1098	1.419	0.2297	1.3909					
0.1943	2.3729	0.1066	0.6098					
0.2708	3.1696	0	0					
0	0	0.5683	5.2656	0.1722	3.5606	0.8692	1.092	2.759
0.1399	2.104	0.2926	2.0621					
0.2496	3.4721	0.1369	0.8922					
0.3562	4.7231	0	0					
0	0	0.8096	17.003	0.2845	6.8179	0.7207	1.333	2.287
0.1854	3.708	0.3877	3.6333					
0.334	5.9063	0.1832	1.5178					
0.4808	7.9057	0	0					

^a^Standard uncertainties *u* for temperature, pressure, mass fraction, and molality are *u(T)* = 0.1 K, *u*(*P*) = 0.01 kPa, *u*(*w*) = 0.002, and *u(m)* = 0.003 mol kg^−1^, respectively with confidence level of 0.95. The combined standard uncertainty of vapor pressure, water activities, and the osmotic coefficient are *u* (*p*) = 0.02 kPa, *u* (*a*_w_) = 0.002, and *u* (*ϕ*_R_) = 0.02, respectively with confidence level of 0.68.

^b^*w*_1_ and *m*_1_ are mass fraction and molality of betaine, respectively.

^c^*w*_2_ and *m*_2_ are mass fraction and molality of PEGDME_250_, respectively.

**Table 4:** Mass fractions (*w*) in isopiestic equilibrium, water activity (*a*_W_), osmotic coefficient (${\phi }_{R}$) and vapor pressure (*p*) for the {betaine (1) + K_3_PO_4_ (2) + H_2_O (3)} system at *T* = 298.15 K and *P* ≈ 85 kPa^a^.

^*b*^*w*_2_	$\frac{{m}_{1}}{{\mathrm{mol}\cdot \mathrm{kg}}^{-1}}$	^*c*^*w*_2_	${m}_{2}/{\mathrm{mol}\cdot \mathrm{kg}}^{-1}$	${w}_{NaCl}$	${m}_{NaCl}/{\mathrm{mol}\cdot \mathrm{kg}}^{-1}$	${a}_{w}$	${\phi }_{R}$	$p/\mathrm{kPa}$
0	0	0.1851	0.8529	0.0731	1.3496	0.9545	0.957	3.03
0.0558	0.5725	0.1115	0.5027					
0.109	1.112	0.0547	0.2455					
0.1872	1.9654	0	0					
0	0	0.2015	0.9476	0.0808	1.5034	0.9491	0.965	3.013
0.0606	0.6325	0.121	0.5553					
0.1182	1.226	0.0593	0.2707					
0.201	2.1478	0	0					
0	0	0.2374	1.1691	0.0965	1.8281	0.9374	0.982	2.976
0.0708	0.7666	0.1412	0.673					
0.1371	1.474	0.0688	0.3254					
0.2293	2.5395	0	0					
0	0	0.3184	1.7544	0.1371	2.7189	0.9035	1.036	2.868
0.0912	1.071	0.182	0.9404					
0.1763	2.046	0.0884	0.4516					
0.3004	3.6655	0	0					
0	0	0.3719	2.2232	0.1626	3.3233	0.8791	1.076	2.791
0.1052	1.3119	0.2101	1.1519					
0.2009	2.456	0.1008	0.542					
0.3447	4.4897	0	0					
0	0	0.4959	3.6935	0.2914	7.3653	0.6942	1.376	2.203
0.1563	2.5085	0.3119	2.2025					
0.2975	4.589	0.1492	1.0129					
0.4738	7.6853	0	0					

^a^Standard uncertainties *u* for temperature, pressure, mass fraction, and molality are *u(T)* = 0.1 K, *u*(*P*) = 0.01 kPa, *u*(*w*) = 0.002, and *u(m)* = 0.003 mol kg^−1^, respectively with confidence level of 0.95. The combined standard uncertainty of vapor pressure, water activities, and the osmotic coefficient are *u* (*p*) = 0.02 kPa, *u* (*a*_w_) = 0.002, and *u* (*ϕ*_R_) = 0.02, respectively with confidence level of 0.68.

^b^*w*_1_ and *m*_1_ are mass fraction and molality of betaine, respectively.

^c^*w*_2_ and *m*_2_ are mass fraction and molality of K_3_PO_4_, respectively.

**Table 5:** Mass fractions (*w*) in isopiestic equilibrium, water activity (*a*_*W*_), osmotic coefficient (${\phi }_{R}$) and vapor pressure (*p*) for {betaine (1) + K_2_HPO_4_ (2) + H_2_O (3)} system at *T* = 298.15 K and *P* ≈ 85 kPa^a^.

^*b*^* w*_1_	${m}_{1}/{\mathrm{mol}\cdot \mathrm{kg}}^{-1}$	^*c*^* w*_2_	${m}_{2}/{\mathrm{mol}\cdot \mathrm{kg}}^{-1}$	${w}_{NaCl}$	${m}_{NaCl}/{\mathrm{mol}\cdot \mathrm{kg}}^{-1}$	${a}_{w}$	${\phi }_{R}$	$p/\mathrm{kPa}$
0	0	0.2427	1.583	0.1054	1.8579	0.9304	0.993	2.954
0.0757	0.5463	0.1391	0.6753					
0.1485	1.596	0.0747	0.5396					
0.2422	2.4562	0	0					
0	0	0.2946	2.3972	0.1086	2.4874	0.9279	0.997	2.946
0.0924	1.0603	0.1698	1.3108					
0.1762	2.118	0.0886	0.5602					
0.2889	3.664	0	0					
0	0	0.35	3.091	0.1403	2.8139	0.8997	1.042	2.856
0.1077	0.1077	0.1979	0.1979					
0.1957	0.1957	0.0984	0.0984					
0.3301	4.206	0	0					
0	0	0.4244	4.2338	0.2359	5.2853	0.7931	1.217	2.517
0.1371	1.9153	0.252	2.3679					
0.2627	3.704	0.1321	1.2346					
0.4165	6.0933	0	0					
0	0	0.4506	4.7088	0.2717	7.1153	0.7063	1.356	2.242
0.144	2.0777	0.2646	2.5686					
0.254	3.508	0.1277	1.1862					
0.4304	6.4491	0	0					
0	0	0.4764	5.2238	0.3303	10.4509	0.5433	1.62	1.724
0.1587	2.4638	0.2916	3.046					
0.2757	4.018	0.1386	1.3587					
0.4555	7.1413	0	0					

^a^Standard uncertainties *u* for temperature, pressure, mass fraction, and molality are *u(T)* = 0.1 K, *u*(*P*) = 0.01 kPa, *u*(*w*) = 0.002, and *u(m)* = 0.003 mol kg^−1^, respectively with confidence level of 0.95. The combined standard uncertainty of vapor pressure, water activities, and the osmotic coefficient are *u* (*p*) = 0.02 kPa, *u* (*a*_w_) = 0.002, and *u* (*ϕ*_R_) = 0.02, respectively with confidence level of 0.68.

^b^*w*_1_ and *m*_1_ are mass fraction and molality of betaine, respectively.

^c^*w*_2_ and *m*_2_ are mass fraction and molality of K_2_HPO_4_, respectively.

The available water activity data for (PEGDME_250_ + H_2_O)^9,18^], (K_3_PO_4_ + H_2_O)^19^ and (K_2_HPO_4_ + H_2_O)^20^ make it possible to check the quality of our water activity data for these binary solutions by comparisons of these data, as shown in Figs. S1-S3 presented in supporting material. These Figures show good agreement of our measurements for these binary systems with the literature values.

The vapor pressure data for binary (betaine + water), (PEGDME_250_ + water), (K_3_PO_4_ + water) and (K_2_HPO_4_ + water) solutions may be used for comparison of the extent of solute–solvent interactions in these binary solutions. For this purpose, the vapor pressure depression values $\Delta p$ were calculated from the corresponding *P* values reported in Tables 3, 4, 5 and vapor pressure for pure water as follows:

4 $$\Delta p = p_{w}^{0} - p$$

**Figure 4:**
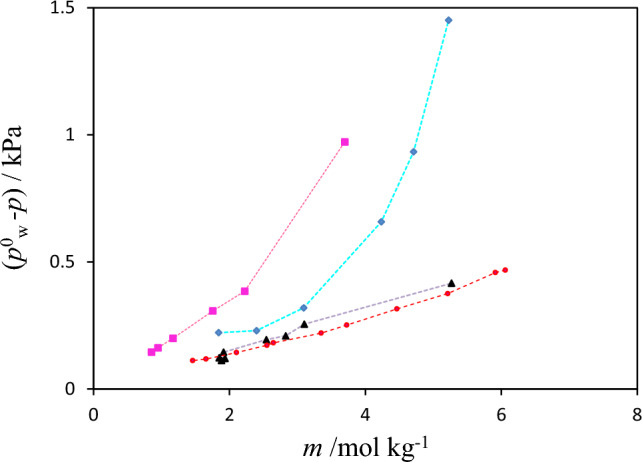
Vapor pressure depression ($\Delta p = p_{w}^{0} - p$) of binary aqueous systems plotted against molality (*m*) of PEGDME_250_, K_3_PO_4_, K_2_HPO_4_ and Betaine at *T* = 298.15 K: “red circle”, {betaine + H_2_O}; “filled triangle”, {PEGDME_250_ + H_2_O}, “pink square”, {K_3_PO_4_ + H_2_O}, “blue diamond”, {K_2_HPO_4_ + H_2_O}.

The plot of calculated vapor pressure depression values for these binary solutions versus molalities of betaine, polymer and salts have been given in Fig. 4. This Figure and Tables 3, 4, 5 show that the vapor pressure depressions for salt solutions are more that of betaine or polymer solutions, and their values for (K_3_PO_4_ + water) are higher than (K_2_HPO_4_ + water) at the same solute concentrations. In other words solute–solvent interactions for salts are stronger than that of betaine or polymer. The differences in interactions between these salt systems are related to their anions; so that, a more vapor pressure depression is observed for the salt which has a higher anion charge (K_3_PO_4_ ˃ K_2_HPO_4_) leading to stronger salt-water interactions. In other words, since vapor pressure depression is a colligative property, its value is increased by increasing the number of moles of ions. The higher $\Delta p$ value observed for aqueous betaine than PEGDME_250_ may be due to the fact that betaine is a hydrogen-bond accepter and PEGDME_250_ is more hydrophobic than the betaine. The more negative logarithm of octanol/water partition coefficient value for betaine (log Kow = − 4.6)^21^ than that the polyethylene glycol (log Kow = − 1.36)^22^, which has similar structure to PEGDME250, indicates lower affinity of PEGDME_250_ to water molecules compared to betaine.

Based on Figs. 1, 2, 3, from the comparison of lines with equal water activity with the line corresponding to semi-ideal solution, the salting-in or salting-out effect in the ternary (betaine + PEGDME_250_ + water), (betaine + K_3_PO_4_ + water) and (betaine + K_2_HPO_4_ + water) systems may be investigated. The Zdanovskii rule was used for this purpose using (Zdanovskii-Stokes-Robinson) relation at constant water activity^23,24^:

5 $$\frac{{m_{1} }}{{m_{1}^{0} }} + \frac{{m_{2} }}{{m_{2}^{0} }} = 1$$

In Eq. (5), $m_{1}$ and $m_{2}$ stand for concentration of PEGDME_250_ or salts and betaine (in molality basis) in the ternary systems, respectively. Similarly, $m_{1}^{0}$ and $m_{2}^{0}$ show respectively, the concentration of the PEGDME_250_ or salts and betaine in the binary solution which has an equal *a*_w_. The results of applying Zdanovskii rule^23^ are also presented in Figs. 1, 2, 3 which indicate that for all the three studied ternary systems the experimental lines with equal water activity indicate negative deviation in regard with semi-ideal behavior. This means that in these investigated ternary systems the interaction between betaine and polymer or salts are unfavorable (salting-out effect). This salting-out effect may be the main reason for phase separation of these systems at some concentrations. Indeed, formation of aqueous two-phase systems (ATPSs) of betaine with both of the salts (K_3_PO_4_ and K_2_HPO_4_) have been observed by Zeng et al.^6^. Also, it was found that betaine can form an ATPS with polyethylene glycol (PEG)^6^. These ATPSs have found important applications in protein extraction^6^. As expected, we observed that betaine can also form an ATPS with PEGDME_250_ which has similar structure with PEG.

### Thermodynamic framework

The polynomial method^10^ has been frequently used for obtaining relation between activity coefficients of solutes and their molalities in ternary systems^25,26^. According to this method, for each component *i* of the ternary system, the activity coefficient in molality basis,${\gamma }_{i}^{m}$, can be expressed in terms of betaine molality, *m*_1_ and salts (K_3_PO_4_/K_2_HPO_4_) or PEGDME_250_ molality, *m*_2_ as follows:

6 $$ln{\gamma }_{i}^{m}=\sum_{i=0}^{\infty }\sum_{j=0}^{\infty }{A}_{ij}{m}_{1}^{i}{m}_{2}^{j} ({A}_{00}=0)$$

Equation 6 can be written as Eq. (7a) when in the power series we neglect all terms higher than fourth:

7a $$ln{\gamma }_{i}^{m}=ln{\gamma }_{i}^{0,m}+{A}_{01}{m}_{1}+{A}_{11}{m}_{1}{m}_{2}+{A}_{02}{m}_{2}^{2}+{A}_{21}{m}_{1}^{2}{m}_{2}+{A}_{12}{{m}_{1}m}_{2}^{2}+{A}_{03}{m}_{2}^{3}+{A}_{31}{m}_{1}^{3}{m}_{2}+{A}_{22}{m}_{1}^{2}{m}_{2}^{2}+{A}_{13}{{m}_{1}m}_{2}^{3}+{A}_{04}{m}_{2}^{4}$$

In Eq. (7a) the term ${\gamma }_{i}^{0,m}$ denotes the activity coefficient of component *i* in aqueous binary solutions which is expressed in terms of its molality, *m*_i_, as follows:

7b $$ln{\gamma }_{i}^{0,m}={A}_{10}{m}_{i}+{A}_{20}{m}_{i}^{2}+{A}_{30}{m}_{i}^{3}+{A}_{40}{m}_{i}^{4}$$

Within this thermodynamic framework a quantity $\Delta$ has been introduced^27^:

8a $$\Delta =-55.51ln{a}_{w}-{m}_{1}{\phi }_{1}^{0}-2{m}_{2}{\phi }_{2}^{0}$$

which is identical with Eq. (8b)

8b $$\Delta =2{m}_{R}{\phi }_{R}^{0}-{m}_{1}{\phi }_{1}^{0}-2{m}_{2}{\phi }_{2}^{0}$$

In above relations, ${\phi }_{R}^{0}, {\phi }_{1}^{0}$, and ${\phi }_{2}^{0}$ are used respectively for the osmotic coefficients of solutions of the reference, betaine, and salts (K_3_PO4/K_2_HPO_4_) or PEGDME_250_.

Then, using the Gibbs–Duhem relation the Eq. (9) can be derived^27^:

9 $$\frac{\Delta }{{m}_{1}{m}_{2}}={A}_{01}+{A}_{11}{m}_{1}+2{A}_{02}{m}_{2}+{3A}_{03}{m}_{2}^{2}+{A}_{21}{m}_{1}^{2}+\left(\frac{3}{2}\right){A}_{12}{m}_{1}{m}_{2}+{A}_{31}{m}_{1}^{3}+\left(\frac{4}{3}\right){A}_{22}{m}_{1}^{2}{m}_{2}+2{A}_{13}{{m}_{1}m}_{2}^{2}+4{A}_{04}{m}_{2}^{3}$$

Using the Eq. 8, the values for $\frac{\Delta }{{m}_{1}{m}_{2}}$ can be easily calculated from the molality values presented in Tables 3, 4, 5 for the reference, betaine, and salts (K_3_PO_4_/K_2_HPO_4_) or PEGDME_250_. Then, the parameters ${A}_{ij}$ in Eq. (9) were determined by the minimization method with the results presented in Tables 6. When these parameters are inserted in Eq. 7, it is easy to calculate concentration dependencies of ${\gamma }_{1}^{m}$ of betaine in solutions composed of salts (K_3_PO_4_/K_2_HPO_4_) or PEGDME_250_.

**Table 6:** The coefficients, ${A}_{ij}$, obtained from Eq. (9) for different systems.

Coefficients	Betaine + PEGDME + H_2_O	Betaine + K_3_PO_4_ + H_2_O	Betaine + K_2_HPO_4_ + H_2_O
${A}_{01}$	− 8.59786	6.30484	1.54286
${A}_{11}$	− 4.01483	− 4.39398	− 0.01862
${A}_{02}$	18.92576	− 6.49056	− 0.01774
${A}_{03}$	− 349.74341	118.30840	11.88388
${A}_{21}$	− 247.64186	68.11242	14.47608
${A}_{12}$	824.63595	− 246.68534	− 37.54101
${A}_{31}$	− 7.65526	− 1.12413	0.13003
${A}_{22}$	18.25862	1.67649	0.01654
${A}_{13}$	5.22736	1.80136	0.01152
${A}_{04}$	− 2.23978	− 3.58109	− 0.00134

To calculate the required ${\phi }_{1}^{0}$ value for betaine and salts (K_3_PO_4_/K_2_HPO_4_) or PEGDME_250_ at each molality *m*_1_ listed respectively in Tables 2 for betaine and Tables 3, 4, 5 (corresponding to zero betaine concentration) for salts (K_3_PO_4_/K_2_HPO_4_) or PEGDME_250_, the obtained corresponding osmotic coefficients were correlated with the Eq. (10):

10 $${\phi }_{i}=1+\sum_{j=1}^{4}{E}_{j}{m}_{i}^{j}$$

Then, the following relation is used for calculation of the activity coefficients of betaine, salts (K_3_PO_4_/K_2_HPO_4_) or PEGDME_250_

11 $$ln{\gamma }_{i}^{0,m}=\sum_{j=1}^{4}\frac{\left(j+1\right)}{j}{E}_{j}{m}_{i}^{j}$$

The symbol *E* denotes the parameters of Eqs. (10) and (11) which can be determined by minimization method.

Using the calculated activity coefficients of betaine in binary and ternary salts (K_3_PO_4_/K_2_HPO_4_) or PEGDME_250_ solutions, the Gibbs energy of transfer,$\Delta {G}_{tr}^{i}$, of betaine from corresponding binary to the ternary solutions were determined from Eq. 12^28^. The sigh of the $\Delta {G}_{tr}^{i}$ may be used to evaluate solute–solute and solute–solvent interactions in the investigated solutions.

12 $$\Delta {G}_{tr}^{i}=RTln\frac{{\gamma }_{i}^{x}}{{\gamma }_{i}^{0x}}$$

${\gamma }_{i}^{x}$ and ${\gamma }_{i}^{0x}$ are respectively mole fraction based activity coefficient of betaine in the ternary and binary solutions. The required ${\gamma }_{i}^{x}$ values were determined from the Eq. (13) using the calculated values of activity coefficients in molality basis:

13 $$ln{\gamma }_{i}^{x}=ln{\gamma }_{i}^{m}+ln{\gamma }_{i}^{\infty }+ln\left(1+\frac{{M}_{w}\nu m}{1000}\right)$$

where ${\gamma }_{i}^{\infty }$ denotes activity coefficient at infinite dilution.

The calculated transfer Gibbs energy values are presented in Table 7. The positive transfer Gibbs energies obtained for all the three mixtures imply unfavorable interaction of betaine with salts (K_3_PO_4_/K_2_HPO_4_) and PEGDME_250_. It is interesting to note that in Sect. 3.1.1 we arrived at the same result regarding these unfavorable interactions by observing the negative deviation of iso-activity lines from semi-ideal solution for the investigated solutions. Table 7 also show that the $\Delta {G}_{tr}^{i}$ of betaine becomes more positive by increasing the concentrations of salts or polymer.

**Table 7:** Gibbs energies of transfer,$\Delta {G}_{tr}^{i}$, of PEGDME_250_, K_3_PO_4_ and K_2_HPO_4_ from water to aqueous betaine solutions at 298.15 K^a^.

^*c*^*m*_2_/ mol⋅kg^−1^	^*b*^m_1_/ mol⋅kg^−1^					
	$$\Delta G_{{tr}}^{i} /J/mol$$					
	Betaine + PEGDME_250_ + H_2_O					
	0.2	0.22	0.24	0.26	0.28	0.3
0.2	1794.1	2365.5	2838.2	3211.9	3486.5	3661.7
0.25	3089.4	4320.9	5429.4	6414.7	7276.5	8014.6
0.3	3420.5	5519.4	7471.3	9275.8	10,932.6	12,441.6

	Betaine + K_3_PO_4_ + H_2_O					
	1.1	1.2	1.3	1.4	1.5	1.6
0.01	7704.2	9108.8	10,637.5	12,289.8	14,065.1	15,962.7
0.02	14,882.8	17,645.1	20,655.7	23,913.6	27,417.4	31,165.9
0.03	21,542.7	25,615.7	30,061.5	34,878.2	40,063.8	45,616.6

	betaine + K_2_HPO_4_ + H_2_O					
	1.1	1.2	1.3	1.4	1.5	1.6
0.01	1867.6	2198.9	2559.9	2950.5	3371	3821.4
0.02	3653.9	4309.2	5023.7	5797.6	6631.1	7524.4
0.03	5359.8	6331.5	7392.1	8541.9	9781	11,109.7

^a^Standard uncertainties *u* for temperature, pressure, and molality are *u(T)* = 0.1 K, *u*(*P*) = 0.01 kPa, and *u(m)* = 0.003 mol kg^−1^, respectively with confidence level of 0.95. The combined standard uncertainty for $\Delta {G}_{tr}^{i}$ is *u*_*c*_*(*$\Delta {G}_{tr}^{i}$*)* = 0.8 J mol^−1^ with confidence level of 0.68.

^b^*m*_1_ is molality of Betaine.

^c^*m*_2_ is molality of PEGDME_250_, or salts (K_3_PO_4_ and K_2_HPO_4_).

Based on McMillan–Mayer theory the obtained $\Delta {G}_{tr}^{i}$ values can also be utilized to get information on the extent of solute–solute interactions in our studied systems^29,30^. According to this theory, at constant temperature and pressure the Gibbs energies of transfer for betaine from water to aqueous salt or polymer solutions are given by Eq. (14):

14 $$\Delta {G}_{tr}^{1}(w\to \,w+salt \,or \,polymer)=2.{\nu }_{1}.{\nu }_{2}.{m}_{1}.{g}_{12}+6.{\nu }_{1}^{2}.{\nu }_{2}.{m}_{1}.{m}_{2}.{g}_{112}+3.{{\nu }_{1}.\nu }_{2}^{2}.{m}_{2}^{2}.{g}_{122}$$

In Eq. (14), *g*_*12*_ is called pair interaction parameter; (*g*_*112*_ and *g*_*122*_) are triplet interaction parameters. These interaction parameters were determined by fitting $\Delta {G}_{tr}^{i}$ data to *m*_*1*_ and* m*_*2*_. From the obtained *g*_*12*_ values and the following relation the salting coefficient ${k}_{s}$ can be calculated:

15 $${RTk}_{s}=2{\nu }_{1}{\nu }_{2}{g}_{12}$$

The pair and triple interaction parameters obtained from Eq. (14) were tabulated in Table 8. Also in this Table salting constant values were included which were calculated from Eq. (15). The positive pair interaction parameter, *g*_*12*_, found for betaine indicates that pairwise interactions between betaine and salts (K_3_PO_4_/K_2_HPO_4_) or PEGDME_250_ are energetically unfavorable. Also the calculated positive salting constant, ${k}_{s}$, tabulated in Table 8 imply that salts (K_3_PO_4_/K_2_HPO_4_) or PEGDME_250_ have the salting-out effect on betaine in these ternary systems. Consequently, the McMillan–Mayer theory also confirms the unfavorable interaction between salts (K_3_PO_4_/K_2_HPO_4_) or PEGDME_250_ and betaine that we concluded in Sect. 3.1.1 from the negative deviation of constant water activity lines with respect to semi-ideal solutions, and from the positive sign of Gibbs energies of transfer for betaine from water to aqueous salts (K_3_PO_4_/K_2_HPO_4_) or PEGDME_250_ solutions discussed above.

**Table 8:** Values of pair (*g*_12_) and triple parameters (*g*_112_ and *g*_122_) together with the salting constant (*k*_s_).

^*a*^*m*_2_/ mol⋅kg^−1^	*g*_12_	*g*_112_	*g*_122_	*k*_s_
	Betaine(1) + PEGDME_250_ (2) + H_2_O(3)			
0.2	2961	2848	− 3701	21,320
0.25	3788.9	11,366	− 8741	27,280
0.3	5319.3	19,149	− 13,312	38,300

	Betaine(1) + K_3_PO_4_(2) + H_2_O(3)			
0.01	1645.3	215	− 1,422,873	29,616
0.02	3245.1	195	− 711,809	58,412
0.03	4776.4	430	− 474,689	58,974

	Betaine(1) + K_2_HPO_4_(2) + H_2_O(3)			
0.01	390.3	14	− 330,681	7026
0.02	773.8	3	− 165,300	13,930
0.03	1140.6	103	− 110,194	20,530

^a^*m*_2_ is molality of PEGDME_250_, K_3_PO_4_ or K_2_HPO_4_.

### Volumetric properties

The experimental density and speed of sound values for binary aqueous betaine and ternary aqueous betaine solutions containing PEGDME_250_ and salts (K_3_PO_4_/K_2_HPO_4_) are collected respectively in Tables 9, 10, 11, 12. No experimental density or speed of sound data for these systems containing betaine have been reported, previously. In Figures S4-S6, the experimental density values for binary aqueous PEGDME_250_, K_3_PO_4_, and K_2_HPO_4_ solutions were compared with the corresponding literature values^31–33^, which show fairly good agreement. The density data presented in Tables 10, 11, 12 were used to calculate values of the apparent molar volumes, $V_{\Phi }$ and the apparent molar volume at infinite dilution, $V_{\Phi }^{0}$ , from which some information regarding intermolecular interactions between betaine and polymer or salts may be deduced. To calculate $V_{\Phi }$ values for betaine in water and ternary {PEGDME_250_ or salt + water} solutions at *T* = 298.15 K the following equation^34^ was used:

16 $$V_{\varphi } = \frac{M}{\rho } - \left( {\frac{{\rho - \rho_{0} }}{{m\rho \rho_{0} }}} \right)$$

here, *M* is the molar mass of betaine and *m* is its molality;* ρ*_0_ and *ρ* stand for density of the (betaine + H_2_O) and ternary mixtures (betaine + PEGDME_250_ + H_2_O) or [(betaine + salts (K_3_PO_4_ and K_2_HPO_4_) + H_2_O], respectively. The obtained $V_{\Phi }$ values are collected in Tables S1–S3 and plotted against betaine molality in Figures S10- S12. It was found that for the investigated systems linear dependence between $V_{\Phi }$ and betaine molality may be established; so that, the following Masson equation^35^ were used to evaluate the values for apparent molar volume at infinite dilution $V_{\Phi }^{0}$:

17 $$V_{\varphi } = V_{\varphi }^{0} + S_{v} m$$

**Table 9:** The values of density (*ρ*) and speed of sound (*u*) for the binary (betaine + water) system at *T* = 298.15 K and *P* ≈ 85 kPa^a^.

^b^*m*/mol kg^−1^	*ρ*.10^–3^/kg m^−3^	*u*/m s^−1^
0.0000	0.997042	1501.78
0.0892	0.998611	1503.33
0.1094	0.998978	1504.94
0.1300	0.999356	1506.45
0.1488	0.999708	1508.93
0.1793	1.000299	1510.57

^a^Standard uncertainties *u* for temperature, pressure, and molality are *u(T)* = 0.01 K, *u*(*P*) = 0.01 kPa, and *u(m)* = 0.003 mol kg^−1^, respectively with confidence level of 0.95. The combined standard uncertainty of density and speed of sound are *u*_*c*_ (*ρ*) = 0.15 kg m^−3^ and *u*_*c*_ (*u*) = 0.9 m s^−1^, respectively with confidence level of 0.68.

^b^*m* is the betaine molality.

**Table 10:** The values of density (*ρ*) and speed of sound (*u*) for the betaine in aqueous (PEGDME_250_ + water) solutions at *T* = 298.15 K and *P* ≈ 85 kPa^a^.

^b^*m*_b_/mol kg^−1^	*ρ*.10^–3^/kg m^−3^	*u*/m s^−1^
*Betaine in [PEGDME*_*250*_* + water (m*_*p*_* = 0.1 mol kg*^*−1*^*)]*^*c*^		
0.0000	0.999246	1511.96
0.0699	1.000470	1516.16
0.0892	1.000808	1517.46
0.1096	1.001164	1518.94
0.1283	1.001490	1520.30
0.1451	1.001783	1521.66
0.1754	1.002308	1524.22
0.1992	1.002719	1526.35
*Betaine in [PEGDME*_*250*_* + water (m*_*p*_* = 0.2 mol kg*^*−1*^*)]*		
0.0000	1.001538	1527.11
0.0699	1.002756	1531.92
0.0892	1.003083	1533.32
0.1096	1.003427	1534.83
0.1283	1.003793	1536.46
0.1451	1.004101	1537.82
0.1754	1.004570	1540.01
0.1992	1.004977	1541.94
*Betaine in [PEGDME*_*250*_* + water (m*_*p*_* = 0.3 mol kg*^*−1*^*)]*		
0.0000	1.003767	1541.55
0.0699	1.004985	1546.59
0.0892	1.005308	1548.00
0.1096	1.005661	1549.56
0.1283	1.006044	1551.26
0.1451	1.006377	1552.76
0.1754	1.006862	1554.99
0.1992	1.007343	1557.22

^a^Standard uncertainties *u* for temperature, pressure, and molality are *u(T)* = 0.01 K, *u*(*P*) = 0.01 kPa, and *u(m)* = 0.003 mol kg^−1^, respectively with confidence level of 0.95. The combined standard uncertainty of density and speed of sound are *u*_*c*_ (*ρ*) = 0.15 kg m^−3^ and *u*_*c*_ (*u*) = 0.9 m s^−1^, respectively with confidence level of 0.68.

^b^*m*_b_ is the betaine molalities dissolved per kg of (PEGDME_250_ + water).

^c^*m*_p_ is the molality of polymer in water.

**Table 11:** The values of density (*ρ*) and speed of sound (*u*) for the betaine in aqueous (K_3_PO_4_ + water) solutions at *T* = 298.15 K and *P* ≈ 85kPa^a^.

^b^*m*_b_/mol kg^−1^	*ρ*.10^–3^/kg m^−3^	*u*/m s^−1^
*Betaine in [K*_*3*_*PO*_*4*_* + water (m*_*K3PO4*_* = 0.1 mol kg*^*−1*^*)*^*c*^*]*		
0.0000	1.019501	1521.40
0.0709	1.020637	1525.99
0.0877	1.020918	1527.39
0.1090	1.021273	1529.14
0.1300	1.021633	1530.96
0.1450	1.021888	1532.25
0.1798	1.022495	1535.29
0.1987	1.022829	1536.96
*Betaine in [K*_*3*_*PO*_*4*_* + water (m*_*K3PO4*_* = 0.2 mol kg*^*−1*^*)]*		
0.0000	1.042385	1545.05
0.0709	1.043389	1550.49
0.0877	1.043660	1552.40
0.1090	1.043958	1554.60
0.1300	1.044309	1557.16
0.1450	1.044580	1559.15
0.1798	1.045008	1562.30
0.1987	1.045277	1564.12
*Betaine in [K*_*3*_*PO*_*4*_* + water (m*_*K3PO4*_* = 0.3 mol kg*^*−1*^*)]*		
0.0000	1.065016	1568.64
0.0709	1.065963	1575.04
0.0877	1.066227	1577.10
0.1090	1.066495	1579.28
0.1300	1.066629	1580.42
0.1450	1.067038	1583.73
0.1798	1.067462	1587.32
0.1987	1.067740	1589.63

^a^Standard uncertainties *u* for temperature, pressure, and molality are *u(T)* = 0.01 K, *u*(*P*) = 0.01 kPa, and *u(m)* = 0.003 mol kg^−1^, respectively with confidence level of 0.95. The combined standard uncertainty of density and speed of sound are *u*_*c*_ (*ρ*) = 0.15 kg m^−3^ and *u*_*c*_ (*u*) = 0.9 m s^−1^, respectively with confidence level of 0.68.

^b^m_b_ is the betaine molalities dissolved per kg of (K_3_PO_4_ + water).

^c^m_K3PO4_ is the molality of K_3_PO_4_ in water.

**Table 12:** The values of density (*ρ*) and speed of sound (*u*) for the betaine in aqueous (K_2_HPO_4_ + water) solutions at *T* = 298.15 K and *P* ≈ 85kPa^a^.

^b^m_b_/mol kg^−1^	*ρ*.10^–3^/kg m^−3^	*u*/m s^−1^
*Betaine in [(K*_*2*_*HPO*_*4*_* + water (m*_*K2HPO4*_* = 0.1 mol kg*^*−1*^*)*^*c*^*]*		
0.0000	1.011812	1512.82
0.0687	1.012880	1516.98
0.0873	1.013157	1518.64
0.1078	1.013451	1520.61
0.1263	1.013715	1522.49
0.1476	1.014007	1524.59
0.1799	1.014437	1527.95
0.1966	1.014652	1529.62
*Betaine in [K*_*2*_*HPO*_*4*_* + water (m*_*K2HPO4*_* = 0.2 mol kg*^*−1*^*)]*		
0.0000	1.026691	1528.63
0.0687	1.028055	1535.68
0.0873	1.028325	1537.78
0.1078	1.028589	1539.98
0.1263	1.028837	1542.02
0.1476	1.029187	1544.98
0.1799	1.029429	1547.11
*Betaine in [K*_*2*_*HPO*_*4*_* + water (m*_*K2HPO4*_* = 0.3 mol kg*^*−1*^*)]*		
0.0000	1.042394	1544.85
0.0687	1.043599	1550.78
0.0873	1.043911	1552.74
0.1078	1.044239	1554.91
0.1263	1.044490	1556.78
0.1476	1.044785	1559.10
0.1799	1.045142	1562.04
0.1966	1.045498	1565.09

^a^Standard uncertainties *u* for temperature, pressure, and molality are *u(T)* = 0.01 K, *u*(*P*) = 0.01 kPa, and *u(m)* = 0.003 mol kg^−1^, respectively with confidence level of 0.95. The combined standard uncertainty of density and speed of sound are *u*_*c*_ (*ρ*) = 0.15 kg m^−3^ and *u*_*c*_ (*u*) = 0.9 m s^−1^, respectively with confidence level of 0.68.

^b^*m*_b_ is the betaine molalities dissolved per kg of (K_2_HPO_4_ + water).

^c^*m*_K2HPO4_ is the molality of K_2_HPO_4_ in water.

**Figure 5:**
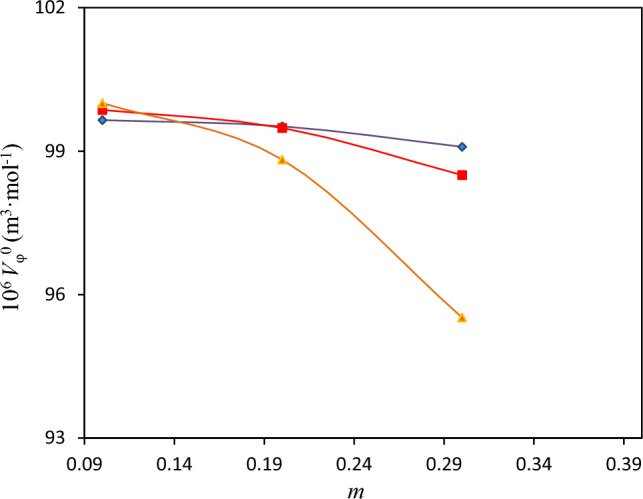
Plot of apparent specific volumes $V_{\Phi }^{0}$ of betaine in aqueous solutions against molality, *m*, of (polymer or salt + water) at *T* = 298.15 K: “blue diamond”, PEGDME_250_; “pink square”, K_3_PO_4_; “yellow triangle”, K_2_HPO_4_.

The values of $V_{\Phi }^{0}$ and empirical slope $S_{V}$ obtained from fitting of $V_{\Phi }$ values to betaine molality are presented in Table 13. In Figures S10—S12 the straight lines generated by Eq. (17) for the three investigated systems indicate validity of Mason equation^35^.The obtained apparent molar volume at infinite dilution $V_{\Phi }^{0}$ for different systems were plotted against molality of (polymer or salt + water) in Fig. 5. It is also quite clear from the Table 13 and Fig. 5 that the $V_{\Phi }^{0}$ values decreased with the increase in molality of (polymer or salt + water) as a solvent and this leads to conclusion that interaction is unfavorable between polymer or salt and betaine. This is consistent with the result of vapor–liquid equilibrium study of the investigated ternary solutions mentioned in section "Result and discussion".

**Table 13:** The obtained values of limiting apparent specific volume ($V_{\varphi }^{0}$), experimental slopes ($S_{V}$), standard transfer volume (Δ_tr_*V*_φ_^0^) and standard deviation for apparent molar volume *σ* (*V*_φ_) for different ternary aqueous betaine solutions at *T* = 298.15 K^a^.

^*b*^m/mol kg^−1^	10^6^ *V*_φ_^0^ (m^3^·mol^−1^)	10^6^ *S*_v_ (m^3^·mol^−1^)	10^6^ Δ_tr_*V*_φ_^0^ (m^3^·mol^−1^)	^*c*^10^6^ *σ* (*V*_φ_) (m^3^·mol^−1^)
*PEGDME*_*250*_				
0.1	99.649	− 1.14	− 0.822	0.01
0.2	99.520	− 1.12	− 0.951	0.00
0.3	99.091	− 1.31	− 1.380	0.01
*K*_*3*_*PO*_*4*_				
0.1	99.862	− 7.04	− 0.609	0.02
0.2	99.487	− 6.94	− 0.984	0.03
0.3	98.499	− 5.95	− 1.972	0.01
*K*_*2*_*HPO*_*4*_				
0.1	100.005	7.00	− 0.466	0.01
0.2	98.823	8.59	− 1.648	0.02
0.3	95.521	11.26	− 4.950	0.03

^a^Standard uncertainties *u* for temperature, pressure, and molality are *u(T)* = 0.01 K, *u*(*P*) = 0.01 kPa, and *u(m)* = 0.003 mol kg^−1^, respectively with confidence level of 0.95.

^b^*m* is the molality of betaine in water.

^c^ $\sigma (V\varphi ) = \sqrt {\frac{{(V_{\varphi }^{\exp } - V_{\varphi }^{cal} )^{2} }}{n}}$, where *n* is the number of experimental data.

The solute- solute interactions in the studied ternary solutions can be deduced from the behavior of apparent molar volumes as plotted against betaine molality in Figures S10-S12. These Figures show that while the slope for the system containing K_3_PO_4_ is negative the corresponding one for the K_2_HPO_4_ is positive. This difference may be due to the more pronounced chemical reaction between betaine and K_3_PO_4_ than K_2_HPO_4_ which leads to decrease of solute–solute interactions in ternary solutions containing betaine and K_3_PO_4_ as indicated with the negative *S*_v_ values reported in Tables 13. The reason for possible reaction between betaine and salts in water is that the used salts hydrolyze in water and form alkaline solution, especially K_3_PO_4_. The betaine is an amphoteric molecule and therefore it can react with the alkaline solution. However, this reaction can be occurred readily with K_3_PO_4_ than K_2_HPO_4_.

The standard partial molar volumes of transfer (Δ_tr_*V*_φ_^0^) is another important thermodynamic property that can be used for investigating the solute–solvent interactions. Here, the Δ_tr_*V*_φ_^0^ is the difference between $V_{\Phi }^{0}$ values of aqueous ternary solutions containing, K_3_PO_4_, K_2_HPO_4_ or PEGDME_250_ and binary aqueous betaine solutions as follows:

18 $$\Delta_{{{\text{tr}}}} V_{\varphi }^{0} = V_{\varphi }^{0} \left( {{\text{in}}\;{\text{aqueous}}\;{\text{ternary}}} \right) - V_{\varphi }^{0} \left( {{\text{in}}\;{\text{aqueous}}\,{\text{binary}}} \right)$$

The $V_{\Phi }^{0}$ for binary aqueous PEGDME_250_, K_3_PO_4_, and K_2_HPO_4_ solutions are available in the literature^31–33^. The obtained $V_{\Phi }^{0}$ value for binary aqueous betaine solution is given Table 13. At infinite dilution solute – solute interactions are unaffected on the value of Δ_tr_*V*_φ_^0^^13,36^. The Δ_tr_*V*_φ_^0^ values for the ternary {betaine + PEGDME_250_ + water}, {betaine + K_3_PO_4_ + water} and {betaine + K_2_HPO_4_ + water} solutions were also reported in Table 13. This Table shows that the Δ_tr_*V*_φ_^0^ values are negative and become more negative at higher concentrations of PEGDME_250_ and both salts. This means that the presence of betaine in mentioned systems lead to strong interactions between polymer or salts and water. In other words, the betaine-polymer and betaine- phosphate salts interactions are unfavorable; and therefore, phase separation in these ternary systems may be occurred at certain concentrations of betaine, polymer or salts. Indeed, in these systems we observed the formation of two-phase systems. Therefore, both the VLE and volumetric studies predict phase separation in the investigated solutions.

### Acoustic properties

The apparent molar isentropic compressibility *κ*_φ_ and its value at infinite dilution *κ*_φ_^0^ are also important quantities which provide some formation regarding the solvent structure around betaine within the bulk solution. The apparent isentropic molar compression for betaine in aqueous solution of (0.1, 0.2, and 0.3 mol kg^−1^) polymer or salts were calculated and tabulated in Table S1–S3 using the Eq. (19)^37^.

19 $$\kappa_{\varphi } = \frac{{(\kappa_{s} \rho_{0} - \kappa_{s0} \rho )}}{{m\rho \rho_{0} }} + \frac{{\kappa_{s} M}}{\rho }$$

The Laplace – Newton's equation^38^ was used for calculation of the required isentropic compressibility *κ*_s_ of mixture:

20 $$\kappa_{s} = \frac{1}{{\rho u^{2} }}$$

For speed of sound *u* and density *ρ*, the measured values presented in Tables 10, 11, 12 were used.

*κ*_s0_ is the isentropic compressibility of pure solvent. The measured speed of sound data obtained for (PEGDME_250_ + H_2_O), (K_3_PO_4_ + H_2_O) and (K_2_HPO_4_ + H_2_O) may be compared with literature data as Figures S7- S9 respectively. On the basis of these Figures we observe that there are fairly good agreement between speeds of sound data obtained in this work and the literature^31–33^. The calculated *κ*_φ_ values reported in Tables S1–S3 indicate that for all the investigated systems their values decrease with increasing of the betaine concentration.

**Figure 6:**
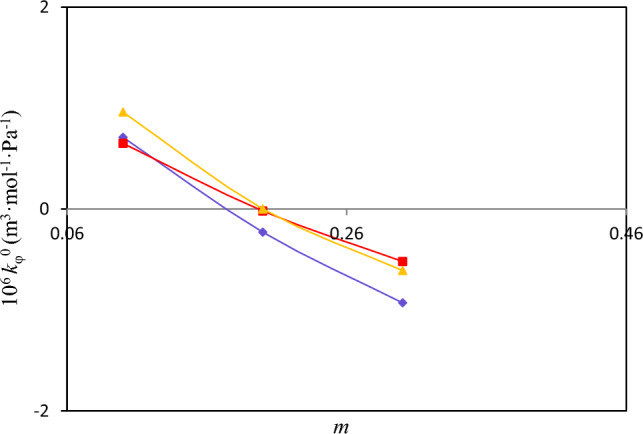
Plot of apparent isentropic compressibility *κ*_φ_^0^ of betaine in aqueous solutions against molality, *m*, of (polymer or salt + water) at *T* = 298.15 K: “blue diamond”, PEGDME_250_; “pink sqaure”, K_3_PO_4_; Yellow triangle”, K_2_HPO_4_.

The apparent molar isentropic compressibility at infinite dilution *κ*_φ_^0^ was also determined from fitting of *κ*_φ_ values to the following relation^38^:

21 $$\kappa_{\varphi } = \kappa_{\varphi }^{0} + S_{\kappa } m$$

here $S_{K}$ is an experimental slope indicative of solute – solvent interactions. The values of *κ*_φ_^0^ and $S_{K}$ together with the standard deviation* σ* (*κ*_φ_^0^) obtained from least square fitting of *κ*_φ_ values to betaine molality *m* are reported in Table 14. The plots of obtained *κ*_φ_ values against betaine molality shown in Figures S13-S15 indicate that *κ*_φ_ values were satisfactorily correlated with Eq. 21.In Fig. 6, the *κ*_φ_^0^ values were plotted against concentration of polymer or salts for different studied systems. It is seen from Table 14 and Fig. 6 that the *κ*_φ_^0^ values for the studied systems decrease with increasing concentration of betaine. Also these values decrease with increase of polymer or salts concentration. The trend observed in variation of $\kappa_{\varphi }^{0}$ or *κ*_φ_ values again implies that while there is a strong interaction between polymer or salts and water, the betaine-polymer and betaine-salts interactions are unfavorable. These results are consistent with the ones we deduced from volumetric studies regarding solute–solvent and solute–solute interactions.

**Table 14:** The obtained values of isentropic compressibility at infinite dilution (*k*_φ_^0^), experimental slopes (*S*_k_), standard transfer isentropic compressibility (Δ_tr_* k*_φ_^0^) and standard deviation for isentropic compressibility* σ* (*k*_φ_) for different ternary aqueous betaine solutions at *T* = 298.15K^a^.

^*b*^m/mol kg^−1^	10^14^ *k*_φ_^0^ (m^3^·mol^−1^·Pa^−1^)	10^14^ *S*_k_ (m^3^·mol^−1^·Pa^−1^)	10^14^ Δ_tr_*k*_φ_^0^ (m^3^·mol^−1^·Pa^−1^)	^*c*^10^14^ *σ* (*k*_φ_) (m^3^·mol^−1^·Pa^−1^)
*PEGDME*_*250*_				
0.1	0.71	− 5.58	− 0.39	0.073
0.2	− 0.23	− 2.26	− 1.33	0.023
0.3	− 0.93	1.02	− 2.03	0.015
*K*_*3*_*PO*_*4*_				
0.1	0.65	− 7.96	− 0.45	0.207
0.2	− 0.02	− 8.54	− 1.12	0.104
0.3	− 0.52	− 5.7	− 1.62	0.094
*K*_*2*_*HPO*_*4*_				
0.1	0.96	− 11.07	− 0.14	0.168
0.2	− 0.00	− 7.74	− 1.10	0.108
0.3	− 0.61	− 5.96	− 1.71	0.088

^a^Standard uncertainties *u* for temperature, pressure, and molality are *u(T)* = 0.01 K, *u*(*P*) = 0.01 kPa, and *u(m)* = 0.003 mol kg^−1^, respectively with confidence level of 0.95.

^b^*m* is the molality of betaine in water.

^c^$\sigma (k\varphi ) = \sqrt {\frac{{(k_{\varphi }^{\exp } - k_{\varphi }^{cal} )^{2} }}{n}}$, where *n* is the number of experimental data.

The transfer molar isotropic compressibility (Δ_tr_*k*_φ_^0^) of betaine from water to aqueous PEGDME_250_ or phosphate salts solutions at infinite dilution were calculated by the help of the following relation:

22 $$\Delta_{{{\text{tr}}}} k_{\varphi }^{0} = k_{\varphi }^{0} \left( {{\text{in}}\;{\text{aqueous}}\;{\text{ternary}}} \right) \, - k_{\varphi }^{0} \left( {{\text{in}}\;{\text{aqueous}}\;{\text{binary}}} \right)$$

For binary aqueous PEGDME_250_, K_3_PO_4_, and K_2_HPO_4_ solutions, the $\kappa_{\varphi }^{0}$ values have been given previously^30–32^. The corresponding $\kappa_{\varphi }^{0}$ value for aqueous betaine is given in Table 14. Similar to transfer apparent molar volume values, the obtained negative Δ_tr_*k*_φ_^0^ values reported in Table 14 indicate strong polymer or salt water interactions implying unfavorable betaine-polymer and betaine-salt interactions which can be regarded as a main reason for biphasic formation of these systems. Therefore, acoustic studies also give the same results as we obtained from volumetric and isopiestic studies regarding the solute–solute interactions in the investigated systems.

## Conclusion

In this work, to investigate a possible interactions between betaine and PEGDME_250_, K_3_PO_4_ or K_2_HPO_4_ in aqueous media, water activity measurements were made on the aqueous systems composed of {betaine + PEGDME_250_}, {betaine + K_3_PO_4_} and {betaine + K_2_HPO_4_} at *T* = 298.15 K and atmospheric pressure by the isopiestic method. The experimental water iso-activity lines showed that these systems have negative deviation from the semi-ideal solutions implying unfavorable interactions between betaine and polymer or salts. This means that these ternary solutions have tendency to form two-phase systems, which was confirmed experimentally. From thermodynamic treatment of water activity data, activity coefficients values for betaine, PEGDME_250_, K_3_PO_4_ and K_2_HPO_4_ in binary and ternary solutions were determined and these values were used to calculate transfer Gibbs energies from binary to ternary solutions. The positive transfer Gibbs energy values obtained for all the studied systems confirm the unfavorable interaction between betaine and PEGDME_250_, K_3_PO_4_ or K_2_HPO_4_. We found further evidence for these interactions by studying volumetric and acoustic properties of the systems by measuring density and speed of sound values. It was found that both of the transfer molar volume ($\Delta_{tr} V_{\varphi }^{0}$) and transfer partial molar isentropic compressibility ($\Delta_{tr} K_{\varphi }^{0}$) for transferring the betaine from water to polymer or salts solutions have negative values; therefore, the same conclusion can be made regarding unfavorable interaction between betaine and polymer or salts and possible phase separation of these ternary solutions as predicted from isopiestic studies. The results obtained in this work serve as a basis for the development of environmentally benign two-phase systems for extraction of drugs and other biomaterials from aqueous media.

### Supplementary Information

Supplementary Information.

## Data Availability

All data generated or analysed during this study are included in this published article [and its supplementary information file].
