# Supplementary material for: Thermodynamic studies of solute–solute and solute–solvent interactions in ternary aqueous systems containing {betaine + PEGDME250} and {betaine + K3PO4 or K2HPO4} at 298.15 K
Source: Sci Rep. 2023 Oct 18;13:17780. doi: 10.1038/s41598-023-43906-0 (PMC10585007; doi:10.1038/s41598-023-43906-0)
Supplement: Supplementary file 1 — Supplementary Information. [file 41598_2023_43906_MOESM1_ESM.docx]

**Thermodynamic studies of solute-solute and solute-solvent interactions in ternary aqueous systems containing {betaine + PEGDME_250_} and {betaine +K_3_PO_4_ or K_2_HPO_4_} at 298.15 K**

**Mohammed Taghi Zafarani-Moattar,**^*^ **Hemayat Shekaari, Soheila Asadollahi**

*Department of Physical Chemistry, University of Tabriz, Tabriz, Iran*

Corresponding author. Tel: +98 4133393135 Fax: 98 4133340191.

E-mail addresses: zafarani47@yahoo.com.

Figure S1. Water activity of (PEGDME_250_+ H_2_O) solutions plotted against molality, *m*, of PEGDME_250_ at *T*=298.15 K: ●, this work; ▲, ref. [18]; ■, ref. [19].

Figure S2. Water activity of (K_3_PO_4_ + H_2_O) solutions plotted against molality, *m*, of K_3_PO_4_ at *T*=298.15 K: ●, this work; ■, ref. [20].

Figure S3. Water activity of (K_2_HPO_4_ + H_2_O) solutions plotted against molality, *m*, of K_2_HPO_4_ at *T*=298.15 K: ●, this work; ■, ref. [21].

[18] R. Sadeghi, Y. Shahebrahimi, Vapor−Liquid Equilibria of Aqueous Polymer Solutions from Vapor-Pressure Osmometry and Isopiestic Measurements, J. Chem. Eng. Data 56 (2011) 789–799. https://doi.org/10.1021/je100178s.

[19] M.T. Zafarani-Moattar, H. Shekaari, F. Ghaffari, Evaluation of solute-solvent interaction and phase separation for aqueous polymers solutions containing choline chloride/D-sucrose natural deep eutectic solvent through vapor-liquid equilibria, volumetric and acoustic studies, J. Chem. Thermodyn. 142 (2020) 105963. https://doi.org/10.1016/j.jct.2019.105963.

[20] M.T. Zafarani-Moattar, S. Sarmad, Osmotic and activity coefficient of 1-ethyl-3-methylimidazolium chloride in aqueous solutions of tri-potassium phosphate, potassium carbonate, and potassium chloride at T=298.15K, Calphad. 35 (2011) 331–341. https://doi.org/10.1016/j.calphad.2011.04.008.

[21] M. Kabiri-Badr, M.T. Zafarani-Moattar, Volumetric and Isopiestic Studies of (H_2_O + K_2_HPO_4_ + KH_2_PO_4_) at 25 degree C, ACS Publications. (2002). https://doi.org/10.1021/je00018a009.

Figure S4. Density of (PEGDME_250_ + H_2_O) solutions plotted versus molality, *m*, of PEGDME_250_ at *T*=298.15: ♦, this work; ■, ref. [32].

Figure S5. Density of (K_3_PO_4_ + H_2_O) solutions plotted versus molality, *m*, of K_3_PO_4_ at *T*=298.15: ♦, this work; ■, ref. [31]; ▲, ref. [34].

Figure S6. Density of (K_2_HPO_4_ + H_2_O) solutions plotted versus molality, *m*, of K_2_HPO_4_ at *T*=298.15: ♦, this work; ■, ref. [33]; ▲, ref. [34].

Figure S7. Speed of sound of (PEGDME_250_ + H_2_O) solutions plotted versus molality, *m*, of PEGDME_250_ at *T*=298.15 K: ■, this work; ■, ref [32].

Figure S8. Speed of sound of (K_3_PO_4_ + H_2_O) solutions plotted versus molality, *m*, of K_3_PO_4_ at *T*=298.15 K: ■, this work; ■, ref [33]; ▲, ref. [34].

Figure S9. Speed of sound of (K_2_HPO_4_ + H_2_O) solutions plotted versus molality, m, of K_2_HPO_4_ at *T*=298.15 K: ■, this work; ■, ref. [31]; ▲, ref. [34].

[32] S. Ebrahimi, R. Sadeghi, Density, speed of sound, and viscosity of some binary and ternary aqueous polymer solutions at different temperatures, J. Chem. Eng. Data 60 (2015) 3132–3147. <https://doi.org/10.1021/acs.jced.5b00290>.

[33] M.T. Zafarani-Moattar, H. Shekaari, P. Ardi-Samberan, Thermodynamic Studies of l-Tryptophan and l-Threonine Partitioning in Aqueous Two-phase Systems Containing Deep Eutectic Solvents (Choline Chloride/PEG) and Potassium Salts, J. Chem. Eng. Data 67 (2022) 1214–1227. <https://doi.org/10.1021/acs.jced.2c00062>.

[34] R. K. Ameta, M. Singh, R. K. Kale, Comparative study of density, sound velocity and refractive index for (water + alkali metal) phosphates aqueous systems at *T* = (298.15, 303.15, and 308.15) K. J. Chem. Thermodyn. 60 (2013), 159–168. <https://doi.org/10.1016/j.jct.2013.01.012>.

Figure S10. Apparent molar volumes of betaine versus its molality, *m*, in the presence of different concentration of K_3_PO_4_: ■, *m*_K3PO4_ = 0 mol kg^-1^, ■, *m*_K3PO4_ = 0.1 mol kg^-1^, ▲, *m*_K3PO4_ = 0.2 mol kg^-1^_,_ and ●, *m*_K3PO4_ = 0.3 mol kg^-1^ at 298.15 K under atmospheric pressure (85 kPa). —, generated from equation 17.

Figure S11. Apparent molar volumes of betaine versus its molality, *m*, in the presence of different concentration of K_2_HPO_4_: ■, *m*_K2HPO4_ = 0 mol kg^-1^, ■, *m*_K2HPO4_ = 0.1 mol kg^-1^, ▲, *m*_K2HPO4_ = 0.2 mol kg^-1^_,_ and ●, *m*_K2HPO4_ = 0.3 mol kg^-1^ at 298.15 K under atmospheric pressure (85 kPa). —, generated from equation 17.

Figure S12. Apparent molar volumes of betaine versus its molality, *m*, in the presence of different concentration of PEGDME_250_: ■, *m*_PEGDME250_ = 0 mol kg^-1^, ■, *m*_PEGDME250_ = 0.1 mol kg^-1^, ▲, *m*_PEGDME250_ = 0.2 mol kg^-1^_,_ and ●, *m*_PEGDME250_ = 0.3 mol kg^-1^ at 298.15 K under atmospheric pressure (85 kPa). —, generated from equation 17.

Figure S13. Isentropic compression of betaine versus its molality, *m*, in the presence of different concentration of K_3_PO_4_: ■, *m*_K3PO4_ = 0 mol kg^-1^, ■, *m*_K3PO4_ = 0.1 mol kg^-1^, ■, *m*_K3PO4_ = 0.2 mol kg^-1^_,_ and ●, *m*_K3PO4_ = 0.3 mol kg^-1^ at 298.15 K under atmospheric pressure (85 kPa). —, generated from equation 21.

Figure S14. Isentropic compression of betaine versus its molality, *m*, in the presence of different concentration of K_2_HPO_4_: ■, *m*_K2HPO4_ = 0 mol kg^-1^, ■, *m*_K2HPO4_ = 0.1 mol kg^-1^, ■, *m*_K2HPO4_ = 0.2 mol kg^-1^_,_ and ●, *m*_K2HPO4_ = 0.3 mol kg^-1^ at 298.15 K under atmospheric pressure (85 kPa). —, generated from equation 21.

Figure S15. Isentropic compression of betaine versus its molality, *m*, in the presence of different concentration of PEGDME_250_: ■, *m*_PEGDME250_ = 0 mol kg^-1^, ■, *m*_PEGDME250_ = 0.1 mol kg^-1^, ■, *m*_PEGDME250_ = 0.2 mol kg^-1^_,_ and ●, *m*_PEGDME250_ = 0.3 mol kg^-1^ at 298.15 K under atmospheric pressure (85 kPa). —, generated from equation 21.

Table S1. The values of apparent specific volume () and apparent specific isentropic compressibility (*κ*_φ_) for betaine in aqueous PEGDME_250_ solutions at *T* = 298.15K.

| ^c^*m*_b_/mol.kg^-1^ | .10^6^/m^3^.mol^-1^ | *κ*_φ_.10^14^/m^3^.mol^-1^.pa^-1^ |
| --- | --- | --- |
| Betaine in (water + PEGDME_250_ (mp=0.1mol.kg^-1^)) | | |
| 0.0687 | 99.58 ± 0.15 | 0.101 ± 0.05 |
| 0.0873 | 99.54 ± 0.15 | 0.004 ± 0.05 |
| 0.1078 | 99.52 ± 0.20 | -0.115 ± 0.05 |
| 0.1263 | 99.49 ± 0.20 | -0.196 ± 0.05 |
| 0.1476 | 99.48 ± 0.30 | -0.303 ± 0.06 |
| 0.1799 | 99.45 ± 0.35 | -0.482 ± 0.08 |
| 0.1966 | 99.43 ± 0.40 | -0.618 ± 0.08 |
| Betaine in (water + PEGDME_250_ (mp=0.2 mol.kg^-1^)) | | |
| 0.0687 | 99.44 ± 0.20 | -0.356 ± 0.06 |
| 0.0873 | 99.42 ± 0.30 | -0.426 ± 0.06 |
| 0.1078 | 99.40 ± 0.30 | -0.489 ± 0.09 |
| 0.1263 | 99.38 ± 0.25 | -0.547 ± 0.09 |
| 0.1476 | 99.35 ± 0.25 | -0.580 ± 0.08 |
| 0.1799 | 99.33 ± 0.25 | -0.651 ± 0.08 |
| 0.1966 | 99.30 ± 0.30 | -0.706 ± 0.09 |
| Betaine in (water + PEGDME_250_ (mp=0.3 mol.kg^-1^)) | | |
| 0.0687 | 99.01 ± 0.30 | -0.564 ± 0.09 |
| 0.0873 | 98.97 ± 0.30 | -0.614 ± 0.09 |
| 0.1078 | 98.95 ± 0.25 | -0.656 ± 0.09 |
| 0.1263 | 98.91 ± 0.25 | -0.693 ± 0.09 |
| 0.1476 | 98.89 ± 0.30 | -0.723 ± 0.08 |
| 0.1799 | 98.86 ± 0.40 | -0.765 ± 0.08 |
| 0.1966 | 98.83 ± 0.35 | -0.801 ± 0.08 |

Table S2. The values of apparent specific volume () and apparent specific isentropic compressibility (*κ*_φ_) for betaine in aqueous K_3_PO_4_ solutions at *T* = 298.15K.

| ^c^mb/mol.kg^-1^ | .10^6^/m^3^.mol^-1^ | *κ*_φ_.10^14^/m^3^.mol^-1^.pa^-1^ |
| --- | --- | --- |
| Betaine in (water + K_3_PO_4_ (m_K3PO4_ = 0.1mol.kg^-1^)) | | |
| 0.0687 | 99.39 ± 0.20 | 0.0130 ± 0.09 |
| 0.0873 | 99.23 ± 0.30 | -0.196 ± 0.09 |
| 0.1078 | 99.10 ± 0.30 | -0.356 ± 0.09 |
| 0.1263 | 98.92 ± 0.40 | -0.508 ± 0.08 |
| 0.1476 | 98.84 ± 0.20 | -0.585 ± 0.08 |
| 0.1799 | 98.60 ± 0.09 | -0.738 ± 0.08 |
| 0.1966 | 98.47 ± 0.25 | -0.806 ± 0.07 |
| Betaine in (water + K_3_PO_4_ (m_K3PO4_ = 0.2 mol.kg^-1^)) | | |
| 0.0687 | 98.97 ± 0.30 | -0.478 ± 0.07 |
| 0.0873 | 98.88 ± 0.35 | -0.757 ± 0.07 |
| 0.1078 | 98.78 ± 0.40 | -1.010 ± 0.07 |
| 0.1263 | 98.61 ± 0.40 | -1.228 ± 0.07 |
| 0.1476 | 98.46 ± 0.40 | -1.376 ± 0.08 |
| 0.1799 | 98.28 ± 0.30 | -1.551 ± 0.08 |
| 0.1966 | 98.14 ± 0.30 | -1.613 ± 0.08 |
| Betaine in (water + K_3_PO_4_ (m_K3PO4_ = 0.3 mol.kg^-1^)) | | |
| 0.0687 | 98.08 ± 0.25 | -0.855 ± 0.09 |
| 0.0873 | 97.96 ± 0.30 | -1.039 ± 0.12 |
| 0.1078 | 97.85 ±0.45 | -1.207 ± 0.11 |
| 0.1263 | 97.80 ± 0.35 | -1.288 ± 0.12 |
| 0.1476 | 97.64 ± 0.36 | -1.448 ± 0.12 |
| 0.1799 | 97.46 ±0.40 | -1.622 ± 0.13 |
| 0.1966 | 97.34 ± 0.25 | -1.708 ± 0.13 |

Table S3. The values of apparent specific volume () and apparent specific isentropic compressibility (*κ*_φ_) for betaine in aqueous K_2_HPO_4_ solutions at *T* = 298.15K.

| ^c^m_b_/mol.kg^-1^ | .10^6^/m^3^.mol^-1^ | *κ*_φ_.10^14^/m^3^.mol^-1^.pa^-1^ |
| --- | --- | --- |
| Betaine in (water + K_2_HPO_4_ (m_K2HPO4_ = 0.1mol.kg^-1^)) | | |
| 0.0687 | 100.50 ± 0.08 | 0.260 ± 0.07 |
| 0.0873 | 100.60 ± 0.07 | -0.077 ± 0.07 |
| 0.1078 | 100.76 ± 0.06 | -0.381 ± 0.07 |
| 0.1263 | 100.88 ± 0.06 | -0.611 ± 0.06 |
| 0.1476 | 101.03 ± 0.08 | -0.778 ± 0.06 |
| 0.1799 | 101.27 ± 0.08 | -1.000 ± 0.09 |
| 0.1966 | 101.38 ± 0.10 | -1.067 ± 0.09 |
| Betaine in (water + K_2_HPO_4_ (m_K2HPO4_ = 0.2 mol.kg^-1^)) | | |
| 0.0687 | 99.58 ± 0.20 | -0.624 ± 0.07 |
| 0.0873 | 99.78 ± 0.09 | -0.882 ± 0.07 |
| 0.1078 | 99.93 ± 0.20 | -1.107 ± 0.06 |
| 0.1263 | 100.11 ± 0.09 | -1.227 ± 0.06 |
| 0.1476 | 100.31 ± 0.25 | -1.361 ± 0.05 |
| 0.1799 | 100.50 ± 0.30 | -1.413 ± 008 |
| Betaine in (water + K_2_HPO_4_ (m_K2HPO4_ = 0.3 mol.kg^-1^)) | | |
| 0.0687 | 96.32 ± 0.15 | -1.032 ± 0.09 |
| 0.0873 | 96.52 ± 0.20 | -1.192 ± 0.09 |
| 0.1078 | 96.70 ± 0.35 | -1.329 ± 0.09 |
| 0.1263 | 96.96 ± 0.30 | -1.421 ± 0.10 |
| 0.1476 | 97.21 ± 0.30 | -1.531 ± 0.11 |
| 0.1799 | 97.49 ± 0.20 | -1.636 ± 0.11 |
| 0.1966 | 97.74 ± 0.40 | -1.722 ± 0.12 |
